# Comprehensive genotyping analysis of single nucleotide polymorphisms responsible for beef marbling in Japanese Black cattle

**DOI:** 10.1186/s12863-024-01199-w

**Published:** 2024-02-09

**Authors:** Shinji Sasazaki, Hina Kondo, Yurika Moriishi, Fuki Kawaguchi, Kenji Oyama, Hideyuki Mannen

**Affiliations:** 1 Laboratory of Animal Breeding and Genetics, Graduate School of Agricultural Science, Kobe University, Kobe, Japan; 2 Food Resources Education & Research Center, Kobe University, Kasai, Japan

**Keywords:** GWAS, Beef marbling, Japanese Black cattle, Meat quality, ICAM1

## Abstract

**Background:**

Beef marbling is considered a desirable trait in the meat industry. Therefore, understanding the genetic factors that cause marbling is important. Previously, we performed a genome-wide association study to examine genetic factors associated with beef marbling in Japanese Black cattle and identified a candidate region between 10–30 Mbp on chromosome 7. We verified the effect of the SNPs in this region on beef marbling using linkage disequilibrium block analysis. We narrowed down the candidate region to a range of 15.8–16.1 Mbp. In this study, we comprehensively detected all of the SNPs in this region and verified their effects on beef marbling.

**Results:**

Genome resequencing using four animals exhibiting high beef marbling standard (BMS) and four with low BMS revealed a total of 1,846 polymorphisms within the candidate region. Based on the annotation, we selected 13 SNPs exhibiting a moderate impact, as no high-impact SNPs were detected. All of the SNPs represented missense polymorphisms and were located in the following seven genes: *RDH8*, *ANGPTL6*, *DNMT1*, *MRPL4*, *ICAM1*, *ICAM3*, and *ICAM5*. Finally, we determined the effects of these SNPs on the BMS of a Japanese Black cattle population (*n* = 529). Analysis of variance revealed that the five SNPs were located in genes encoding the intercellular adhesion molecules (*ICAM1*, *ICAM3*, and *ICAM5*), and showed a highly significant association compared with the remainder (*p* < 0.01). The lowest p-value was observed for ICAM3_c.739G > A (*p* = 1.18E-04). Previous studies have suggested that intercellular adhesion molecules (ICAM) may be an upstream factor that regulates adipocyte differentiation. Therefore, considering the polymorphism and putative gene function, we suggest that *ICAM1* is potentially responsible for beef marbling. c.470C > G and/or c.994G > A on ICAM1 may be responsible for this quantitative trait locus.

**Conclusions:**

Promising SNP candidates responsible for beef marbling were identified using extensive polymorphism verification in a previously reported QTL region. We aim to elucidate the mechanism of beef marbling in future studies by investigating how these polymorphisms alter protein structure and function.

**Supplementary Information:**

The online version contains supplementary material available at 10.1186/s12863-024-01199-w.

## Background

In the beef industry, marbling is an important feature for assessing meat quality. Japanese Black cattle, a predominant breed in Japan, are highly valued for their rich, marbled meat, which is the result of past breeding and improvement efforts. Recent studies indicate that beef marbling is a highly heritable trait in these cattle (0.4–0.6), suggesting the untapped potential for further improvement [1, 2]. Previous attempts to identify genes and polymorphisms associated with beef marbling revealed several relevant polymorphisms. For example, a polymorphism in the promoter region of the endothelial differentiation gene 1 (*EDG1*) gene, which is involved in blood vessel formation [3], was strongly associated with beef marbling in Japanese Black cattle, thus making it a candidate gene [4–6]. However, this polymorphism was not significantly associated with beef marbling in all Japanese Black cattle populations [7]. Moreover, considering the location of this polymorphism, it may affect the expression of the *EDG1* gene; however, there is currently no evidence to support this hypothesis. Therefore, further studies are needed to identify the underlying polymorphisms and their corresponding genes.

As beef marbling may be regulated by multiple genes, several studies searching for candidate genes have been done using genome-wide association analysis (GWAS). Although several candidate regions and genes have been implicated, the precise genes involved have not yet been identified [8–10]. This may be due to a lack of functional information for these genes, which makes it challenging to select candidate genes within a specific region. The mechanism of beef marbling formation is likely very complex and functional information regarding the genes involved in this process may be unknown. Therefore, candidate genes should be identified using an approach that does not strongly depend on the functional information to elucidate the relevant genes.

In our previous study, a GWAS on beef marbling was performed on a Japanese Black cattle population and a potential locus spanning 10–30 Mbp on chromosome 7 was identified [11]. This locus was implicated as a Quantitative Trait Locus (QTL) for beef marbling in diverse cattle populations and breeds other than Japanese Black cattle [12–14]. We then used genomic resequencing to identify 127,090 polymorphisms in this region. Of these, we selected 96 SNPs as candidates for further validation based on gene annotation and linkage disequilibrium (LD) using the most significant SNPs (i.e., the "top" SNPs) from the GWAS [15]. These SNPs were genotyped using a Dynamic Array integrated fluidic circuit using two prefectural Japanese cattle populations, Hyogo and Miyazaki, and their effects on beef marbling were evaluated [16]. In the Hyogo population, the exact area of the LD block could not be determined as the LD structure was strong throughout this region. However, in the Miyazaki population, we identified an LD block containing SNPs that were significantly associated with beef marbling within the 15.8–16.1 Mbp region, which suggested a candidate region. In the present study, we focused on the Miyazaki population and comprehensively identified the polymorphisms within this candidate region and verified their effects on beef marbling.

## Methods

### Animals

All experiments were carried out according to the Kobe University Animal Experimentation Regulations. We used a Japanese Black cattle population bred in the Miyazaki prefecture, which consisted of 529 cattle (477 steers and 52 heifers) produced from six sires. The cattle with an average age of 29.1 ± 1.62 months were slaughtered. The average BMS scores in the Miyazaki populations were 6.08 ± 1.96. We extracted genomic DNA from 50 mg of longissimus cervicis muscle samples using a standard phenol–chloroform method. This study did not require ethical review or approval as the samples were collected from cattle that were already slaughtered for sale.

### Whole-genome resequencing

We selected eight out of 529 animals for whole-genome resequencing (WGS) based on their BMS values and the presence of ICAM1_c.994G > A, which was the most significant SNP as demonstrated in previous studies [16] (Table S1). We selected four cattle with a higher BMS (7–10) with the GG genotype and four with a lower BMS (2–3) with the AA genotype. Genome sequencing was performed on these animals using the HiSeq X Five Sequencing System (Illumina Inc., San Diego, CA, USA) and the data were normalized using Genedata Expressionist 9.1.4a. The reads were mapped to the cattle reference genome assembly (UCSC bosTau8) from the UCSC Genome Browser assembly (https://genome-asia.ucsc.edu/cgi-bin/hgGateway) using BWA-MEM 0.7.12. We excluded PCR duplicates using Picard 2.2.4. GATK 3.6 (2016–12-08-g1c2527f) was used to call polymorphisms by comparing the sequenced genomes with the reference genomes. Using SnpEff v4.2 [17], the polymorphisms were annotated to the reference gene sequence (bosTau8) (NCBI RefSeq) based on location (intron, exon, untranslated region, upstream, downstream, splice site, and intergenic region) and characteristics (synonymous/nonsynonymous amino acid replacement, gain/loss of start/stop site, and frameshift mutations) (Table S2). Using the “annotation impact” estimated by SnpEff v4.2　as an index for predicting the effects on the proteins for each polymorphism, we selected candidate polymorphisms. In “annotation impact”, polymorphisms were categorized as “high”, “moderate”, “low”, and “modifier” in order of descending impact.

### Genotyping the candidate polymorphisms

We selected 11 candidate polymorphisms from all detected polymorphisms and they were genotyped using the Kompetitive Allele Specific PCR (KASP) assay and PCR-Restriction Fragment Length Polymorphism (PCR–RFLP) method. Table S3 lists the primer sequences and restriction enzymes. The KASP assay and PCR amplifications were performed according to the manufacturer’s instructions (LGC Genomics, Hoddesdon, Herts, UK).

### LD block analysis

The LD coefficients (r^2^) between polymorphisms were calculated using HAPLOVIEW 4.0 with default settings [18].

### Statistical analysis

We used a general linear model to determine the effects of genotyped SNPs on BMS. The analytical model for the Miyazaki population included the effect of sire, sex, year and month of slaughter, genotype, and linear and quadratic covariates for the age at slaughter. *P*-values were adjusted for multiple testing with Bonferroni correction. The differences between the least-squares mean for genotypes within a gene were assessed using Tukey–Kramer's honestly significant difference (HSD) test.

## Results

### Identification and selection of candidate polymorphisms

Genome resequencing was performed using eight animals (four each with high and low BMS) to comprehensively search for all polymorphisms within the previously identified candidate region (15,819,376–16,114,288 bp). A comparison of the genome sequences of these eight animals with a reference genome sequence revealed a total of 1,846 polymorphisms. The annotation impact was used as an index to select candidate polymorphisms. Of these SNPs, 14 were categorized as “MODERATE”, 39 as “LOW”, and 1,793 as “MODIFIER”. “MODERATE” SNPs included “missense_variant”. “LOW” SNPs included “synonymous_variant” and “splice_region_variant”. “MODIFIER” SNPs included “intron_variant”, “intergenic_region”, “3(5)_prime_UTR_variant” and “downstream(upstream)_variant”. Of the 14 “MODERATE” SNPs, 13 were selected as candidate polymorphisms, excluding one SNP that was monomorphic in all eight Japanese Black cattle (Table 1). All of them were missense polymorphisms and were located in the following seven genes: *RDH8*, *ANGPTL6*, *DNMT1*, *MRPL4*, *ICAM1*, *ICAM3*, and *ICAM5*.

**Table 1:** Thirteen candidate SNPs associated with beef marbling detected using genome-resequencing on BTA7

Position	SNP ID	Gene	SNP	Amino acid substitution	Genotype			GLM *p*-value	GLM Bonferroni-adjusted *p*-value
					AA	AB	BB		
15,820,556	rs720528091	*RDH8*	c.451G > A	V151M	439	87	3	6.19E-01	8.04E + 00
15,820,574	rs136794063	*RDH8*	c.469G > T	A157S	229	253	47	3.96E-02	5.15E-01
15,884,198	rs208565993	*ANGPTL6*	c.368C > T	A123V	349	160	20	1.15E-02	1.49E-01
15,884,496	rs378436650	*ANGPTL6*	c.70G > A	A24T	250	224	55	1.73E-01	2.25E + 00
15,934,431	rs210537571	*DNMT1*	c.1030 T > A	S344T	390	130	9	4.08E-01	5.31E + 00
15,998,765	rs209256672	*MRPL4*	c.136C > A	P46T	434	93	2	2.85E-01	3.70E + 00
16,048,450	rs207869084	*ICAM1*	c.470C > G	A157G	172	290	67	3.45E-04	4.48E-03
16,049,219	rs209971703	*ICAM1*	c.872G > C	S291T	311	198	20	1.13E-02	1.47E-01
16,049,421	rs110207241	*ICAM1*	c.994G > A	A332T	179	297	53	3.33E-04	4.33E-03
16,049,750	rs800810449	*ICAM1*	c.1193A > G	Q398R	471	58	0	9.39E-01	1.22E + 01
16,057,414	rs209660548	*ICAM5*	c.1065G > C	E355D	179	281	69	5.28E-04	6.86E-03
16,088,911	rs110709663	*ICAM3*	c.739G > A	D247N	200	254	75	1.18E-04	1.54E-03
16,089,483	rs110165710	*ICAM3*	c.494A > G	Q165R	204	258	67	4.73E-04	6.15E-03

### Effect of genotyped SNPs on BMS in the Miyazaki population

**Fig. 1:**
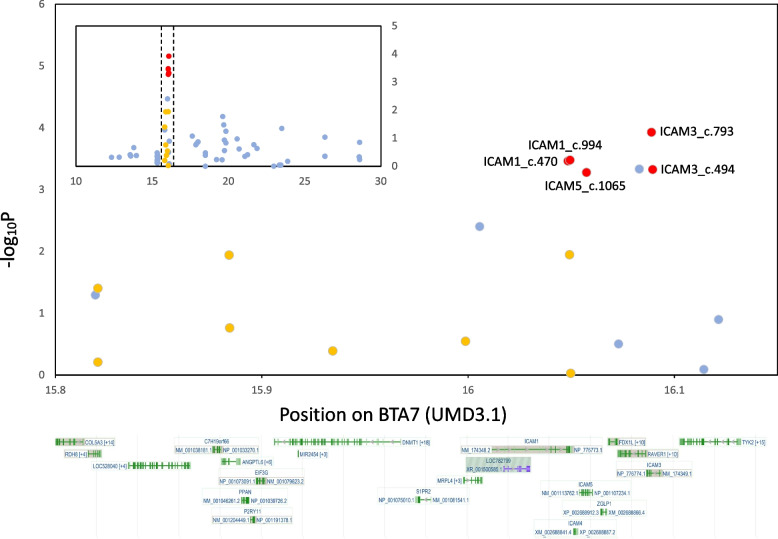
Significance plot of SNPs in the candidate region (15.8–16.1 Mbp on BTA7) and their gene locations. The plot shows the distribution of all the SNPs located in the candidate regions. The x-axis indicates chromosome 7 positions in Mbp and the y-axis indicates log–inverse p-values. The blue circles indicate 76 SNPs that were previously identified in the candidate region (Sasazaki et al., 2022). The red and yellow circles indicate 13 candidate SNPs responsible for beef marbling identified in this study

Among the 13 candidate polymorphisms, the effect of two, ICAM1_c.994G > A and ICAM1_c.1065G > C, was already verified in a previous study using a Dynamic Array integrated fluidic circuit [16]. Therefore, the remaining 11 SNPs were genotyped using KASP and PCR–RFLP assays in the Miyazaki population (*n* = 529). The association of 13 candidate polymorphisms with BMS in the Miyazaki population (*n* = 529) was examined and the results are shown in Table 1. Figure 1 shows a significance plot of 76 SNPs, including 65 previously genotyped SNPs [16]. Among the 13 SNPs, the polymorphism with the lowest p-value was ICAM3_c.739G > A (*p* = 1.18E-04). Furthermore, five SNPs located in *ICAM1*, *ICAM3*, and *ICAM5* were significantly associated with BMS at Bonferroni-corrected significance level of *p* < 0.01. We performed Tukey–Kramer's honestly significant difference (HSD) test to verify the allele substitution effect of these five SNPs (Table 2). For ICAM3_c.739G > A, which had the lowest p-value, the genotype frequencies for GG, GA, and AA were 0.142, 0.480, and 0.378, respectively. The allelic frequencies were G = 0.38 and A = 0.62. The least-square means of BMS values for the GG, GA, and AA types were 6.36 (GG), 6.41 (GA), and 5.65 (AA), respectively. These values were significantly different (*p* < 0.05).

**Table 2:** Effect of five significant SNPs on beef marbling score in a Japanese Black cattle population (*n* = 529)

SNP	Genotype frequency (n)			Allelic frequency		BMS (least square mean)		
						± SE		
ICAM1 c.470C > G	CC	CG	GG	C	G	CC	CG	GG
	0.127 (67)	0.548 (290)	0.325 (172)	0.40	0.60	6.144^ab^	6.371^a^	5.629^b^
						± 0.271	± 0.171	± 0.201
ICAM1 c.994G > A	GG	GA	AA	G	A	GG	GA	AA
	0.100 (53)	0.561 (297)	0.338 (179)	0.38	0.62	6.049^ab^	6.371^a^	5.640^b^
						± 0.298	± 0.169	± 0.200
ICAM5 c.1065G > C	GG	GC	CC	G	C	GG	GC	CC
	0.130 (69)	0.531 (281)	0.338 (179)	0.40	0.60	6.204^ab^	6.357^a^	5.643^b^
						± 0.269	± 0.172	± 0.200
ICAM3 c.739G > A	GG	GA	AA	G	A	GG	GA	AA
	0.142 (75)	0.480 (254)	0.378 (200)	0.38	0.62	6.360^a^	6.412^a^	5.650^b^
						± 0.265	± 0.175	± 0.191
ICAM3 c.494A > G	AA	AG	GG	A	G	AA	AG	GG
	0.127 (67)	0.488 (258)	0.386 (204)	0.37	0.63	6.361^a^	6.380^a^	5.684^b^
						± 0.275	± 0.175	± 0.192

^a,^^b^means with different superscripts indicate significant differences between genotypes

### Linkage Disequilibrium (LD) block analysis

**Fig. 2:**
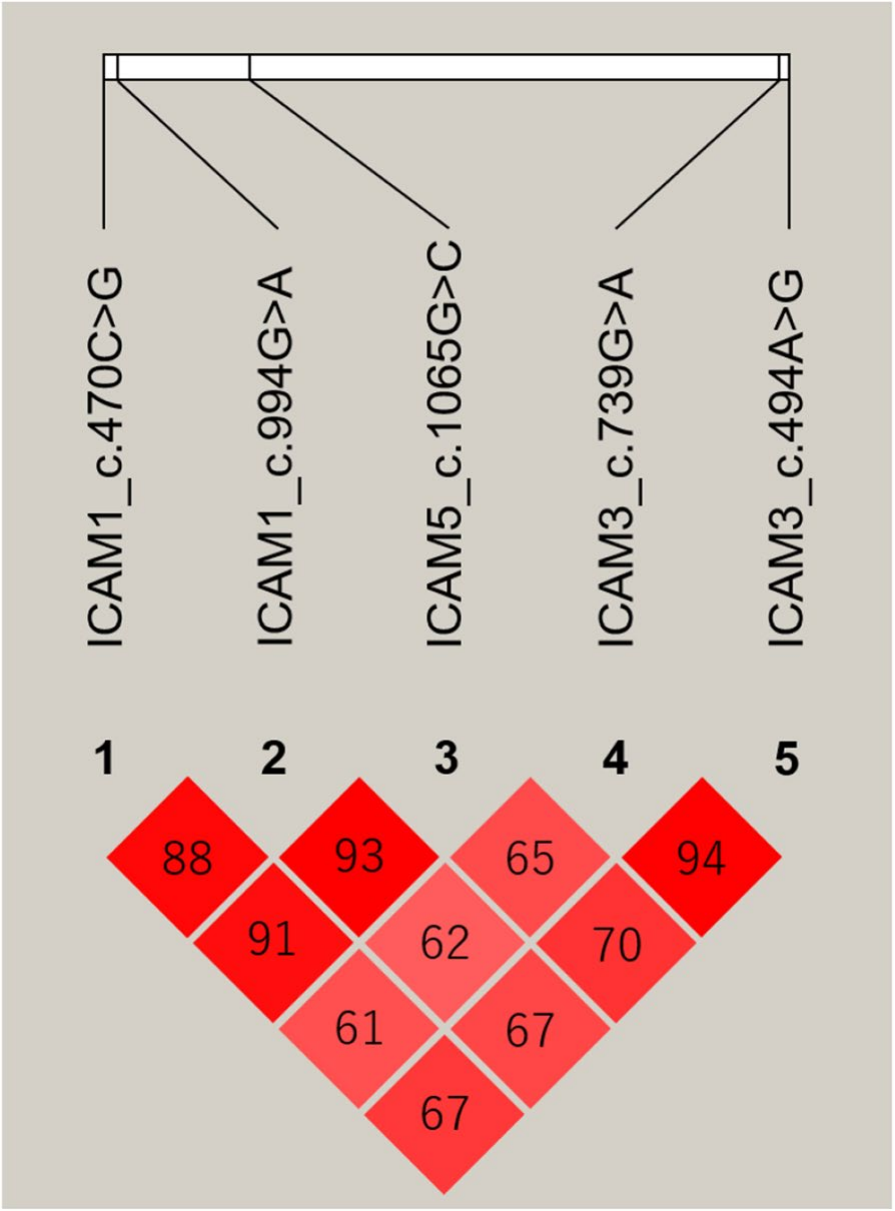
Linkage disequilibrium among the five candidate polymorphisms for beef marbling. Linkage disequilibrium analysis was performed to evaluate the LD coefficients (r^2^) between the five SNPs. One square refers to LD level (r^2^) between two SNPs and the squares are colored by D’/LOD

To assess the independence of the five highly significant SNPs, using the genotyping results of the Miyazaki population (*n* = 529), the linkage disequilibrium coefficients between these SNPs were calculated by Haploview. The highest r^2^ value was observed between the ICAM3_c.739G > A and ICAM3_c.494A > G (r^2^ = 0.94) (Fig. 2). An r^2^ value greater than 0.6 was observed between all SNP pairs.

### Investigation of gene function within the candidate region

We investigated the functional annotation of all 19 genes located within the candidate region using the NCBI gene database (https://www.ncbi.nlm.nih.gov/gene/). Table S4 shows the summary description of the function of each gene. Considering the function of each gene, it seems that no genes are directly involved in lipid metabolism.

## Discussion

Five SNPs located in the *ICAM1*, *ICAM3*, and *ICAM5* genes exhibited a very strong association (*p* < 0.01) compared with the other SNPs, which implicates them in beef marbling. Beef marbling may be genetically improved in this population using these polymorphisms as selection markers. However, we observed a high LD between the five SNPs (r^2^ = 0.61–0.94), suggesting that their effects may not be additive. Moreover, SNPs with a high LD tend to exhibit similar p-values in an association analysis, thus it is difficult to infer a functional effect from the p-value alone. In other words, it is not possible to determine which of these five SNPs functionally affects beef marbling based only on the *p*-value. Therefore, functional analyses, such as evaluating structural changes in each protein, is necessary to identify the responsible polymorphism. Moreover, as multiple SNPs have been implicated, studying their interaction may provide additional information.

Our findings indicate that the five SNPs in genes encoding intercellular adhesion molecules (ICAMs) may be responsible for beef marbling. The ICAMs belong to the immunoglobulin superfamily and five members (ICAM1, 2, 3, 4, and 5) have been identified in various mammals, including cattle. These ICAMs have Ig-like domains and are greater than 50% identical at the amino acid sequence level [19, 20]. Although they have important roles in the immune system [21–23], they are differentially expressed in tissues. ICAM3 and ICAM5 exhibit limited expression in lymphocytes [24] and the terminal brain [25], respectively, whereas ICAM1 is expressed in several tissues and cells, including lymphocytes, endothelial cells, and epithelial cells [26, 27]. In addition to its role in the immune system, ICAM1 mediates cell interactions, promotes leukocyte migration, and regulates inflammatory responses, epithelial injury-resolution responses, and tumorigenesis [28, 29]. ICAM1 is also involved in adipocyte differentiation. Another study showed that it is highly expressed in the preadipocyte stage during the differentiation of human mesenchymal stem cells (MSCs) into adipocytes, suggesting that it influences adipocyte differentiation [30]. Furthermore, an in vitro analysis indicated that overexpression of ICAM1 in MSCs activates extracellular signal-regulated kinase and p38 MAP kinase (p38) [31], which can promote the expression of adipogenic genes through C/EBPβ, which in turn, promotes the differentiation of preadipocytes into adipocytes [32, 33]. Thus, ICAM1 may be an upstream factor regulating adipocyte differentiation and contributing to adipogenesis.

ICAM1 is a transmembrane protein with five extracellular Ig-like domains, which are arranged end-to-end with stabilizing disulfide bonds at conserved cysteine residues [34]. The major ligands, LFA-1 and MAC-1, bind ICAM1 through glutamic acid residues in the Ig-like domains to activate intracellular signaling in the physiological processes described above [35, 36]. In this study, we detected two candidate polymorphisms, c.470C > G and c.994G > A, that were missense polymorphisms and located in the Ig-like domains of ICAM1. Moreover, a missense polymorphism in the Ig-like domain was detected in the human triggering receptor expressed on myeloid cells 2 (TREM2) gene [37]. Its mutant type reduced TREM2 shedding, thereby resulting in the absence of the gene function. As the shedding has also been observed in ICAM1 [38], the two candidate polymorphisms may have functional effects by reducing shedding.

The positions of each missense polymorphism in the Ig-like domain may also provide some insight into their effects on ICAM1 gene function. In the KIT proto-oncogene, receptor tyrosine kinase (KIT) gene, which contains the Ig-like domain as does the ICAM1 gene, missense polymorphisms at or near conserved cysteine residues were strongly associated with human piebaldism, thus suggesting that this region can play a critical role in gene function [39]. Thus, polymorphisms at or near conserved cysteine residues that form a disulfide bond are likely to have a major impact on the structure of Ig-like domains, thereby affecting gene function. c.470C > G caused an amino acid substitution at the 23rd position away from 134th cysteine, whereas c.994G > A caused an amino acid substitution at the 332nd position, which is only two amino acids away from the 330th cysteine that forms a disulfide bond in ICAM1. Additionally, c.994G > A may have a relatively high effect on the tertiary structure of ICAM1 because it substituted a hydrophilic amino acid (alanine) with a hydrophobic amino acid (threonine). These results suggest that c.994G > A may have greater effect on ICAM1 structure than c.470C > G. Further investigations are required to demonstrate such an alteration of ICAM1 structure by missense polymorphisms.

We identified 19 genes in the candidate region (15,819,376-16114288 bp of BTA7) and evaluated their function using databases and other resources. However, no other gene, besides *ICAM1*, was found to be functionally associated with lipid metabolism, which indicates that *ICAM1* is the most promising gene for this QTL from a functional point of view. Therefore, we suggest that the *ICAM1* gene may be responsible for beef marbling in terms of polymorphism effect and gene function. Furthermore, c.470C > G and/or c.994G > A in ICAM1 are likely to be the responsible polymorphisms for this QTL.

## Conclusion

We identified the putative five polymorphisms responsible for beef marbling by verifying candidate SNPs, which were previously found to exhibit a strong QTL in different breeds. We plan to elucidate the mechanism underlying how these polymorphisms alter protein structure and function and their effect on beef marbling.

### Supplementary Information

**Additional file 1: Table S1.** Eight animals used for whole-genome resequencing. **Table S2.** genome resequencing data by comparison among eight animals with a reference genome sequence. **Table S3.** Genotyping method of candidate polymorphsims for beef marbling. **Table S4.** All 19 genes located within the candidate region (15819376–16114288bp) on BTA7.

## Data Availability

No datasets were generated or analysed during the current study.
